# Supplementary material for: Seasonal dynamics in leaf litter decomposing microbial communities in temperate forests: a whole-genome- sequencing-based study
Source: PeerJ. 2024 Sep 23;12:e17769. doi: 10.7717/peerj.17769 (PMC11426322; doi:10.7717/peerj.17769)
Supplement: Figure S1 [file peerj-12-17769-s001.pdf]

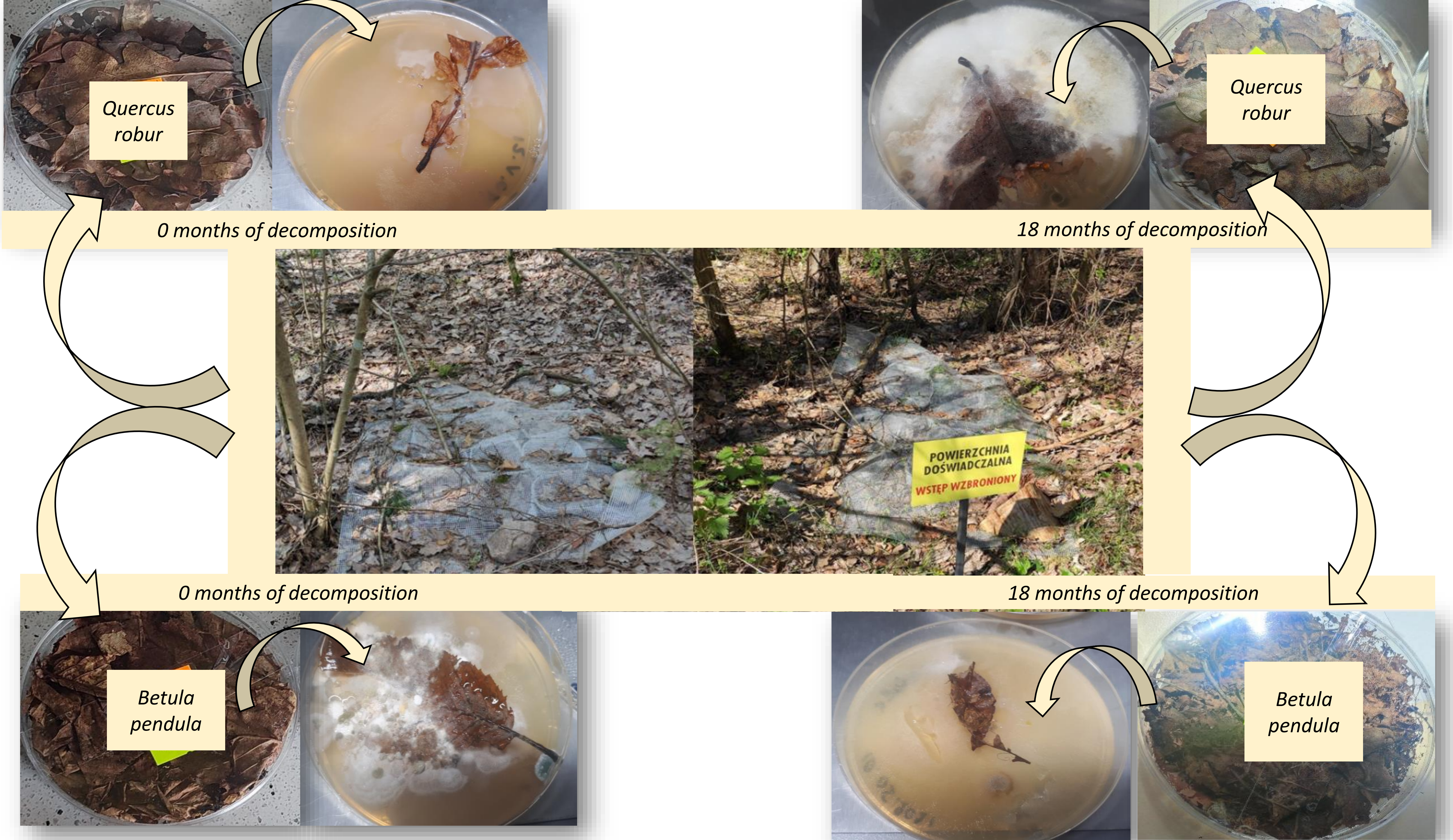
Quercus robur
0 months of decomposition
Quercus robur
18 months of decomposition
POWIERZCHNIA DOŚWIADCZALNA
WSTĘP WZBRONIONY
0 months of decomposition
Betula pendula
18 months of decomposition
Betula pendula
