# Supplementary figures and images for: Seasonal dynamics in leaf litter decomposing microbial communities in temperate forests: a whole-genome- sequencing-based study
Source: PeerJ. 2024 Sep 23;12:e17769. doi: 10.7717/peerj.17769 (PMC11426322; doi:10.7717/peerj.17769)

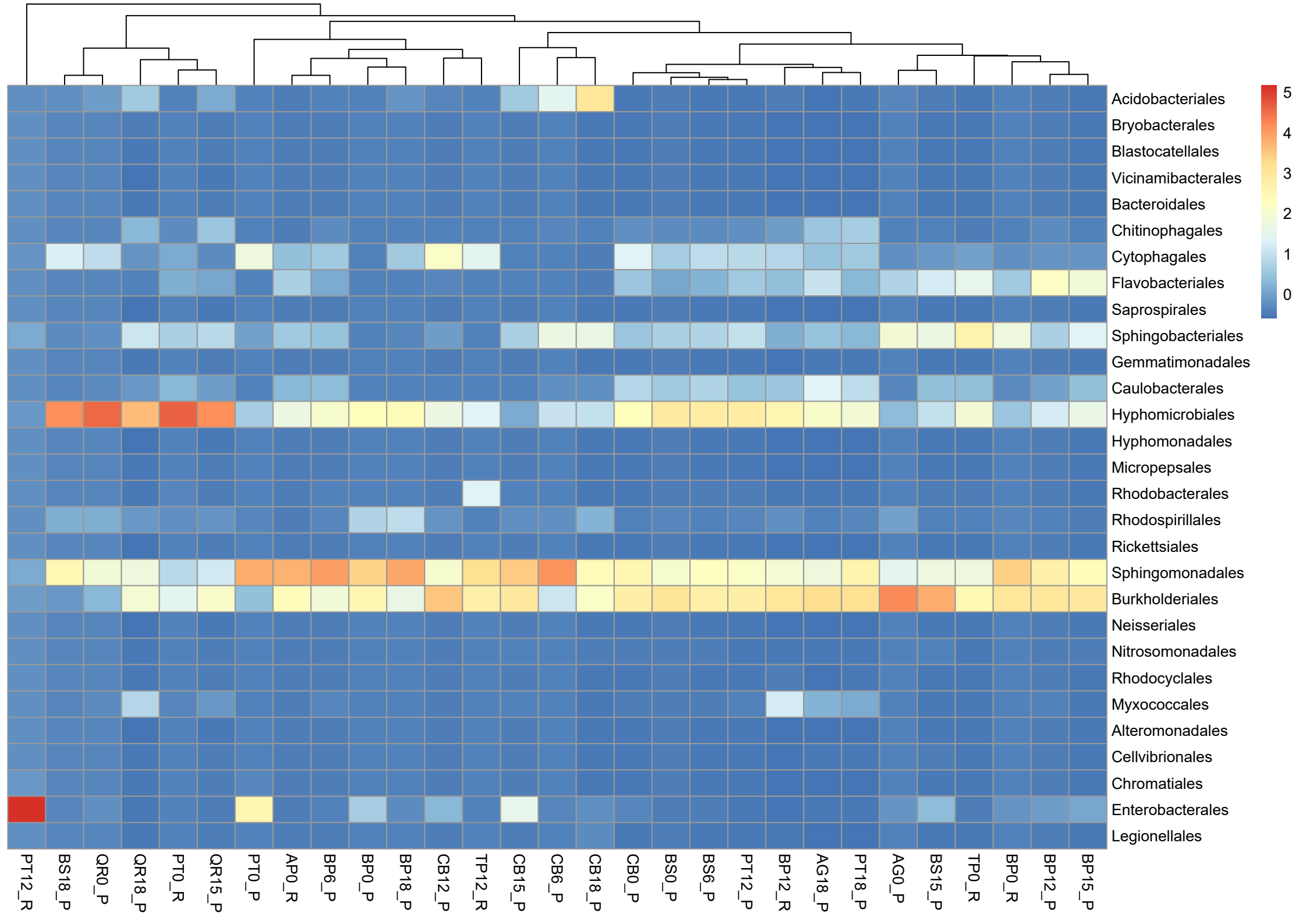

Supplement: Figure S2 [file peerj-12-17769-s002.pdf]

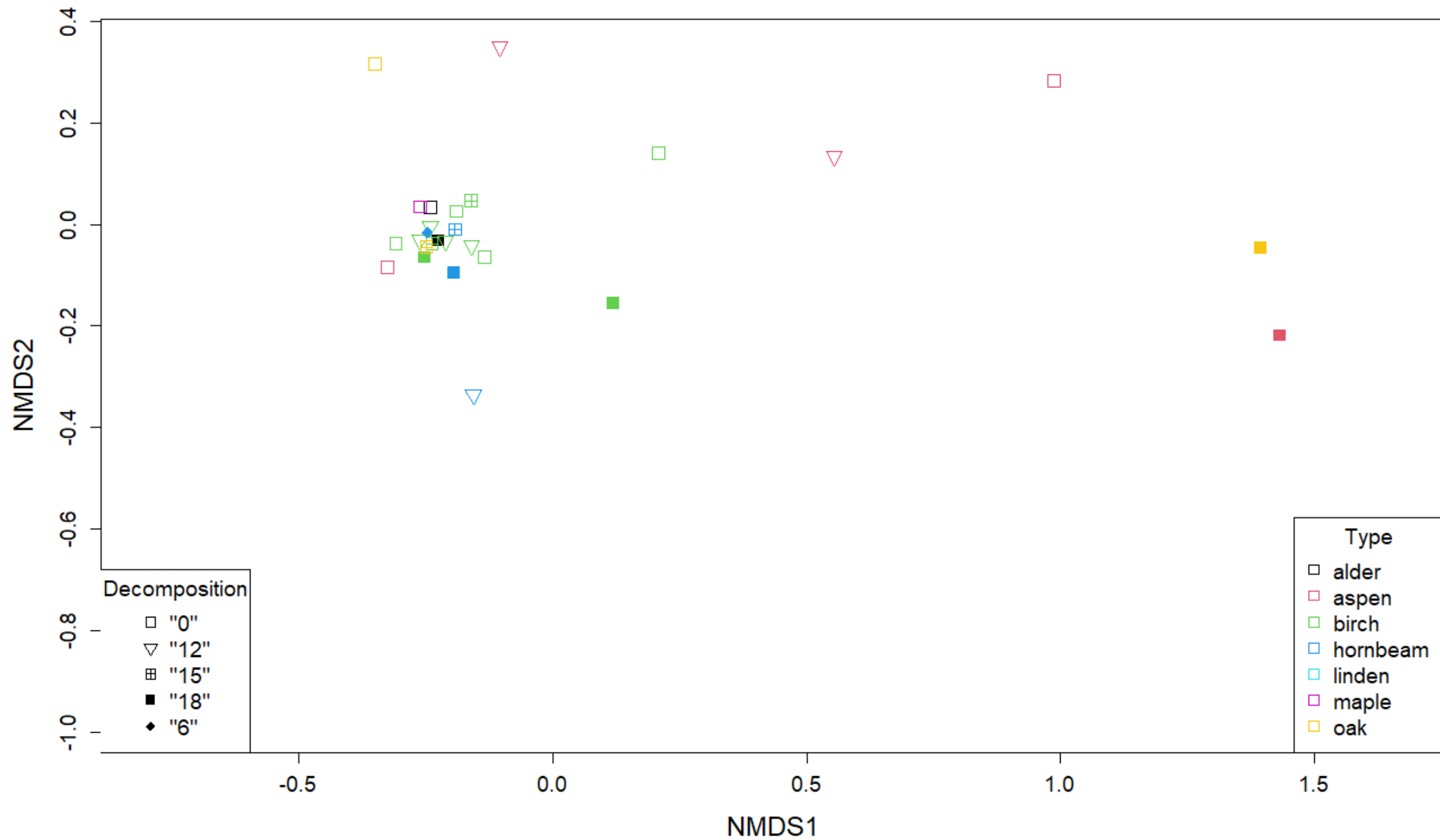

Supplement: Figure S3 [file peerj-12-17769-s003.pdf]

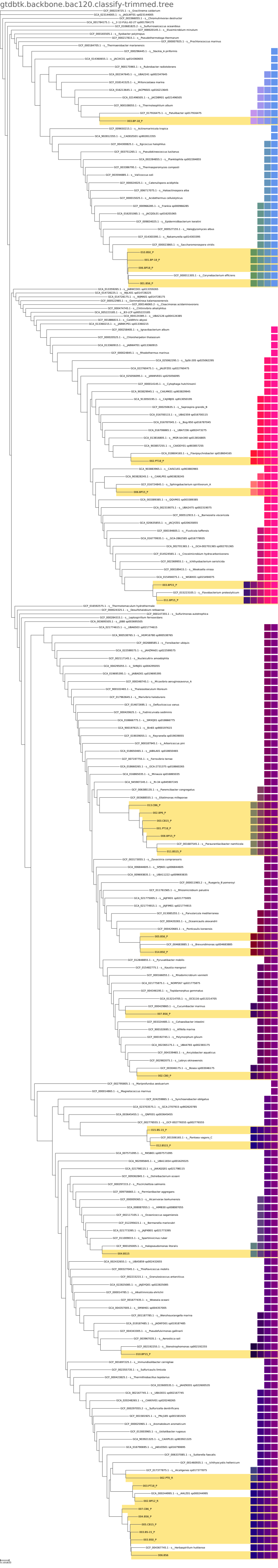

Supplement: Figure S4 [file peerj-12-17769-s004.pdf]
