# Supplementary material for: Seasonal dynamics in leaf litter decomposing microbial communities in temperate forests: a whole-genome- sequencing-based study
Source: PeerJ. 2024 Sep 23;12:e17769. doi: 10.7717/peerj.17769 (PMC11426322; doi:10.7717/peerj.17769)
Supplement: Table S2 [file peerj-12-17769-s006.docx]

**Table S1.** Shannon diversity index calculated for entire microbial communities and description of studied samples.

| Sample name | Shannon | Localization | Type of leaf litter |
| --- | --- | --- | --- |
| AG0_P | 5.7 | Poland | alder |
| AG18_P | 6.6 | Poland | alder |
| AP12_R | 1.8 | Russia | maple |
| BP0_P | 2.4 | Poland | birch |
| BP6_P | 6.1 | Poland | birch |
| BP0_R | 5.8 | Russia | birch |
| BP12_R | 6.2 | Russia | birch |
| BP18_P | 5.1 | Poland | birch |
| BP12_P | 6.5 | Poland | birch |
| BP15_P | 6.3 | Poland | birch |
| BS0_P | 6.4 | Poland | birch |
| BS6_P | 6.5 | Poland | birch |
| BS15_P | 6.22 | Poland | birch |
| BS18_P | 6.6 | Poland | birch |
| CB0_P | 3.3 | Poland | hornbeam |
| CB6_P | 5.8 | Poland | hornbeam |
| CB15_P | 4.7 | Poland | hornbeam |
| CB12_P | 4.6 | Poland | hornbeam |
| CB18_P | 6.2 | Poland | aspen |
| PT0_P | 3.6 | Poland | aspen |
| PT12_P | 6.5 | Poland | aspen |
| PT0_R | 5.7 | Russia | aspen |
| PT12_R | 3.3 | Russia | aspen |
| PT18_P | 6.3 | Poland | aspen |
| QR0_P | 2.7 | Poland | oak |
| QR15_P | 6.3 | Poland | oak |
| QR18_P | 6.1 | Poland | oak |
| TP0_R | 3.9 | Russia | linden |
| TP12_R | 6.2 | Russia | linden |
